# Editorial Materials: Special Issue on Advances in Luminescent Materials

**DOI:** 10.3390/ma19030605

**Published:** 2026-02-04

**Authors:** Luís Pinto da Silva

**Affiliations:** Chemistry Research Unit (CIQUP), Institute of Molecular Sciences (IMS), Department of Geosciences, Environment and Spatial Plannings, Faculty of Sciences, University of Porto, Rua do Campo Alegre s/n, 4169-007 Porto, Portugal; luis.silva@fc.up.pt

Engineered materials are purposely developed and manufactured materials that can be organic, inorganic, or organometallic [1,2,3]. Such materials have attracted significant attention due to their improved performance [4,5], including light emission via down- or upconversion luminescent pathways when excited by UV, visible, or infrared light. More specifically, they have been investigated in various fields, such as imaging [6,7], sensing [8,9], catalysis [10,11], energy storage [12,13], and nanomedicine [14,15].

This Special Issue presents significant advances in various types of luminescent engineered materials, with contributions ranging from the development of practical applications in catalysis and sensing to insights into their photoluminescent properties and an assessment of fabrication strategies. This diversity of contributions highlights the growing importance and broad appeal of luminescent materials to the research community. More specifically, the Special Issue features seven articles, one review paper, and one communication, authored and reviewed by experts in their given fields. This Special Issue presents valuable contributions to the research and development of luminescent engineered materials.

Fernandes et al. [16] investigated if high-yield fabrication strategies for carbon dots could lead to higher yields without higher environmental costs. To that end, a life cycle assessment (LCA) study was conducted to compare the impact of high-yield strategies and “standard” bottom-up procedures, including common precursors (citric acid and glucose) and N-dopants (urea). Standard bottom-up procedures (hydrothermal, microwave-assisted, and thermal treatment) can produce both hydrochar and water-soluble carbon dots, which are considered the desired products but are obtained in lower yields. High-yield approaches focus on the hydrochar obtained and its alkaline peroxide treatment to produce CDs in greater amounts. Relevantly, Fernandes et al. [16] found that high-yield strategies did not generate much lower environmental impacts than “standard” approaches, despite higher yields, indicating that this increase in performance is based on relatively relevant environmental costs.

Kumar et al. [17] synthesized Er^3+^/Yb^3+^-doped/co-doped NaGdF_4_ upconversion nanoparticles via thermal decomposition. More relevantly, multifunctional luminescence-like upconversion and cathodoluminescence were observed under 980 nm laser and electron beam excitation. Furthermore, the particles were used in a non-contact temperature-sensing application over a wide temperature range, from 300 to 1173 K. Thus, this novel material showed distinct luminescence properties and potential for temperature sensing.

Here, Takebuchi et al. [18] investigated the scintillation properties of eulytite-type Ba_3_RE(PO_4_)_3_ (RE = Y, La, and Lu) single crystals, which were synthesized by the floating zone method. The crystals showed a luminescence peak due to self-trapped excitation at about 400 nm under vacuum UV and X-ray irradiation, with the X-ray-induced scintillation decay time constants being several microseconds at room temperature. Interestingly, the luminescence wavelength at about 400 nm is advantageous from the viewpoint of compatibility with the spectral sensitivity of general photodetectors. The scintillation light yields of crystals were quantitatively clarified for the first time, providing relevant information about this type of material.

By their turn, Smith et al. [19] showed that Cd_3_P_2_ quantum dots possess substantial trap emission with radiative times higher than 10^1^ s, and that surface passivation through shell growth or coordination of Lewis acids can accelerate NIR emission. This is due to a decrease in the amount of trap emission. This finding is helpful for future applications of colloidally synthesized quantum dots as quantum emitters, with potential applications in telecommunications and biological imaging.

Kurudirek et al. [20] investigated the effects of additives used in the growth of ZnO nanostructures on their morphological, optical, and scintillation properties. It was found that the addition of sodium citrate significantly reduced defects and increased the intrinsic near-band-edge UV emission intensity at ~380 nm. They also found that the annealing in the forming gas atmosphere enhanced the emission of the UV peak by reducing defects in the nanostructure. The potential of this material to be used as an alpha particle scintillator was also confirmed. Finally, the authors concluded that when ammonium hydroxide and sodium citrate were used, ZnO nanoarrays with improved optical and scintillation properties could be obtained.

To expand the use of CO_2_ capture methodologies, it is important to develop economic incentives for managing the captured CO_2_. One possibility is the production of value-added heterocyclic carbonates from CO_2_ and epoxides. There has been an effort to develop organocatalytic systems for CO_2_ conversion at ambient temperature, composed of a nucleophile and a hydrogen bond donor (HBD). These systems are focused on catalyzing the epoxide-ring opening reaction, which is typically considered the rate-limiting step of the process [21]. To this end, Crista et al. [21] investigated the potential of carbon dots as the HBD component of an organocatalytic system to catalyze epoxide ring-opening at ambient conditions. The rationale for this study is that, in addition to the carbon core, carbon dots have a functionalized surface that may contain hydroxyl and amine groups. The presence of such groups indicates that carbon dots can indeed act as HBDs. The potential of the carbon dots was investigated as an HBD in a model system that used propylene oxide as the target epoxide and pyridine as the nucleophile. Investigation of the epoxide ring-opening reaction showed that carbon dots had better catalytic performance than alternative molecular catalysts, with a rate constant enhancement of 32.2% and a reactant conversion of 70.8%. Therefore, this study indicates the potential of carbon dots as catalysts for CO_2_ utilization strategies, given their promising performance as co-catalysts in the ring-opening reaction of epoxides (the rate-limiting step in a relevant CO_2_ conversion strategy).

Yan [22] reviewed the negative thermal quenching (NTQ) phenomenon of photoluminescence from a macroscopic point of view. NTQ has been reported for a large number of phosphors and is observed when the integral emission intensity of a phosphor increases with increasing temperature, up to a certain elevated level. In this work, the author focused on three points: whether the NTQ of a phosphor is reproducible, whether the data for an NTQ-exhibiting phosphor are consistent with the law of conservation of energy, and whether the NTQ of a phosphor can be demonstrated in prototype WLED devices.

Ion implantation in diamond crystals can be used to produce conducting microstructures and to create isolated photon emitters in quantum optics, photonics, cryptography, and biosensorics. Here, Khomich et al. [23] studied the photoluminescence of helium-related optical centers in diamonds. The authors demonstrated that helium ion implantation in diamonds, followed by annealing, results in several centers in the range of 530–630 nm. The spectral shades of phonon sidebands were determined for helium-related bands 1, 2, and 3, with zero-phonon lines at ~536, 560, and 577 nm. These bands were attributed to interstitial-related centers in diamonds. Thus, the authors provided relevant information for better understanding the properties and structure of helium-related defects in diamonds.

Finally, Shilov et al. [24] investigated the thermal quenching of the intrinsic photoluminescence in amorphous and monoclinic HfO_2_ nanotubes, synthesized by electrochemical oxidation. The authors found that two independent channels of nonradiative relaxation exist, and that optical centers based on vacancies at the 3-fold coordinated oxygen site prevail. This insight is useful for future development of light-emitting devices and thermo-optical sensors based on oxygen-deficient hafnia nanotubes.

In summary, these works made valuable progress in our understanding of different luminescent materials and identified or suggested new avenues of application. Looking forward, the development of luminescent materials faces relevant challenges. For such materials to achieve broader technological and commercial impact, strategies for scalable and reproducible fabrication must be established. Sustainability should also be a focus from the outset, with materials designed to minimize environmental and health impacts, and favor the circular economy. At the same time, safety and reliability remain critical, particularly for applications in biological, optoelectronic, and high-energy systems. Addressing these aspects will not only enhance the practical applicability of luminescent materials but also ensure that their development aligns with long-term environmental and societal goals.

I would like to express my sincere thanks to all authors for their valuable contributions, to the reviewers for their expertise and time, and to the editorial team of *Materials* for their support. Without them, this Special Issue would not have been possible.
